# Cetuximab in preoperative treatment of rectal cancer – term outcome of the XERT trial

**DOI:** 10.2478/v10019-012-0030-2

**Published:** 2012-05-24

**Authors:** Vaneja Velenik, Janja Ocvirk, Irena Oblak, Franc Anderluh

**Affiliations:** 1 Department of Radiotherapy, Institute of Oncology Ljubljana, Ljubljana, Slovenia; 2 Department of Medical Oncology, Institute of Oncology Ljubljana, Ljubljana, Slovenia

**Keywords:** rectal cancer, radio-chemotherapy, cetuximab

## Abstract

**Background:**

Preoperative capecitabine-based chemoradiotherapy (CRT) is feasible for the treatment of resectable locally advanced rectal cancer (LARC). To try to improve efficacy, we conducted a phase II study in which the epidermal growth factor receptor-targeting monoclonal antibody cetuximab was added to capecitabine-based CRT. The results for long-term survival and for an analysis investigating the relationship between survival and patient and disease characteristics, including tumour *KRAS* mutation status, and surgery type, are presented.

**Patients and methods.:**

Patients with resectable LARC received capecitabine (1250 mg/m^2^ twice daily, orally) for 2 weeks followed by cetuximab alone (400 mg/m^2^ for 1 week) and then with CRT (250 mg/m^2^/week) comprising capecitabine (825 mg/m^2^ twice daily) and radiotherapy to the small pelvis (45 Gy in 25 1.8-Gy fractions), five days a week for five weeks. Surgery was conducted six weeks following CRT, with post-operative chemotherapy with capecitabine (1250 mg/m^2^ twice daily for 14 days every 21 days) three weeks later.

**Results:**

Forty-seven patients were enrolled and 37 underwent treatment. Twenty-eight of the patients (75.7%) had T3N+ disease. Thirty-six patients were evaluable for efficacy. The median follow-up time was 39.0 months (range 5.0--87.0). The three-year local control, disease-free survival, relapse-free survival and overall survival rates were 96.9% (95% CI 90.0--100), 72.2% (57.5--86.9), 74.3% (95% CI 59.8--88.8) and 68.1% (95% CI 36.7--99.4), respectively. There was no significant association between survival and gender, age, tumour location in the rectum, type of surgery, pathological T or N status, tumour regression grade or tumour *KRAS* mutation status, although sample sizes were small.

**Conclusions:**

Preoperative cetuximab plus capecitabine-based CRT was feasible in patients with resectable LARC and was associated with an impressive three-year local control rate. The use of tumour *KRAS* mutation status as a biomarker for the efficacy of cetuximab-based regimens in this setting requires further investigation.

## Introduction

Preoperative concurrent chemoradiotherapy (CRT) is associated with improved local tumour control compared with preoperative radiotherapy^1^,^2^ and is a commonly used approach for the treatment of stage II/III locally advanced rectal cancer (LARC). Since the initial observation of the clinical advantages of CRT over radiotherapy, methods of further improving outcomes have been investigated.

In a previous phase II study, we reported that the use of preoperative capecitabine-based CRT was feasible for the treatment of resectable LARC.^3^ We then attempted to improve the efficacy of this regimen by adding the epidermal growth factor receptor (EGFR)-targeting monoclonal antibody, cetuximab. The rationale for the use of this combination was based on the radiosensitizing effects observed with cetuximab in *in vivo* model systems^4^,^5^, supported by clinical evidence from a randomized phase III trial investigating the combination of cetuximab and radiotherapy in the treatment of locally advanced squamous cell carcinoma of the head and neck.^6^,^7^ In the phase III trial, the combination of cetuximab and radiotherapy was significantly more beneficial, in terms of both locoregional control and survival, than radiotherapy alone.

In 2007, we designed a prospective, non-randomized, open label phase II study to investigate the impact of adding cetuximab to preoperative capecitabine-based CRT for the treatment of 37 patients with resectable LARC. The primary endpoint of the trial was pathological complete response (pCR). Results reported in 2010 showed a pCR of 8% (3 patients) with overall-, T- and N-downstaging rates of 73%, 57% and 81%, respectively. The total sphincter preservation rate was 76%.^8^

The identification of biomarkers to tailor treatment to patients most likely to benefit has become an integral part of the investigation of novel treatments and regimens.^9^,^10^ Retrospective analyses of data from randomized trials demonstrated significant improvements in survival when cetuximab was added to standard chemotherapy regimens for the treatment of patients with metastatic CRC not harbouring *KRAS* mutations.^11^,^12^ While the reported incidence rates of tumour *KRAS* mutations in rectal cancer are lower than those for CRC, with rates ranging between 13% and 48%^13^–^17^ compared with the 55–70% reported for metastatic colorectal cancer (mCRC), the presence of such mutations may still have a significant impact on treatment outcome.

We report here the results of the long-term follow-up of our phase II study^8^, with three-year survival results, together with findings from an analysis conducted to investigate any relationship between survival and baseline patient and disease characteristics, including tumour *KRAS* mutation status, and the type of surgery conducted.

## Patients and methods

Details of the study design, eligibility criteria, treatment and assessments have been reported in detail previously.^8^ The study was approved by the relevant ethics committees and was conducted in accordance with the Declaration of Helsinki. It was registered at ClinicalTrials.gov (NCT00689702). All patients provided written informed consent.

### Patients and study design

Briefly, patients with histologically-confirmed International Union Against Cancer (UICC) stage II/III adenocarcinoma of the rectum and a World Health Organization (WHO) performance status (PS) of ≤2 and who had not previously received radiotherapy and/or chemotherapy for their disease were included in the study.^8^ The extent of locoregional disease was determined by magnetic resonance imaging (MRI).

Patients were scheduled to receive eight weeks of treatment, at the end of which the primary tumour was re-evaluated with pelvic MRI and response evaluated according to Response Evaluation Criteria In Solid Tumours (RECIST). Definitive surgery was scheduled to take place four to six weeks after the completion of CRT. A decision on the type of surgery to be conducted was taken prior to the start of preoperative CRT. Three cycles of postoperative chemotherapy, each lasting three weeks, were recommended to start within six weeks of surgery. After the operation the post-treatment surveillance was regularly done.^18^

### Treatment

Capecitabine (1250 mg/m^2^ twice daily orally) was administered for two weeks, followed, on day 1 of week 3, by cetuximab IV (400 mg/m^2^). Starting on day 1 of week 4, patients received cetuximab (250 mg/m^2^/week) plus CRT with capecitabine (825 mg/m^2^ twice daily) and radiotherapy to the small pelvis (45 Gy in 25 1.8-Gy fractions), five days a week for five weeks. Radiation was delivered using three-dimensional conformal computed tomography (CT)-based treatment planning. The chemotherapy component of CRT was initiated on the first day and finished on the last day of radiotherapy (including weekends). Surgery was to be sphincter-sparing where possible. Post-operative chemotherapy comprised capecitabine (1250 mg/m^2^ twice daily for two weeks every three weeks days).

### Assessments

During treatment patients were monitored weekly for safety. Tumour response was assessed five weeks after the end of preoperative treatment. After surgery, histological examination of excised tissue was performed to determine the degree of pathological tumour response. Histological regression of the primary tumour was evaluated using a previously described tumour regression grading (TRG) system: TRG 0=no regression, TRG 1=minimal regression, TRG 2=moderate regression, TRG 3=good regression and TRG 4=total regression.^19^,^20^

### Tumour KRAS mutation analysis

Tumour tissue for *KRAS* analysis was obtained prior to neoadjuvant treatment and at surgery. After microdissection, intratumoural gene expression levels and analysis of seven *KRAS* mutations (codon 12 and codon 13) was carried out using a proprietary procedure, the ResponseDX: Colon™ system (Response Genetics Inc., Los Angeles, California, USA) referred to in previous publications.^21^,^22^

### Statistical methods and considerations

The primary endpoint of the study was the pathological complete response (pCR) rate (defined as the complete disappearance of all tumour cells in surgically excised tissue), results for which have been reported in a separate publication.^8^ Secondary endpoints reported here are: locoregional control (defined as the time from inclusion to documentation of tumour recurrence in the pelvis. It is emphasized that the development, or progression, of metastatic disease did not constitute local failure), relapse-free survival (defined as the time from inclusion to the first occurrence of disease relapse [local or distant], death or date of last follow-up), disease-free survival (defined as the time to relapse, second cancer, or death from any cause, whichever came first) and overall survival (defined as time from inclusion to the date of death from any cause or to the date of last follow-up).

The Kaplan-Meier method was used to estimate the rates of overall, relapse-free, disease-free and local relapse-free survival. A univariate analysis was performed to investigate any association between relapse-free and disease-free survival and parameters including gender, age, tumour location in the rectum (low, middle, upper third), type of surgery (abdominoperineal amputation, sphincter sparing), pathological T or N status, TRG and tumour *KRAS* mutation status. The log-rank test was used to test the significance between the subgroups for this endpoint. The cumulative incidence approach was used to estimate the rates for disease-specific mortality, local recurrence and distant metastasis. Statistical analysis was performed using the SPSS statistical software package, version 12 (SPSS Inc., Chicago, IL, USA).

## Results

### Patients

Between February 2007 and September 2008, 43 patients were recruited into the study. Thirty-seven of these patients subsequently received preoperative treatment with cetuximab plus capecitabine-based CRT. Median age was 55 years, 81.1% were males, and 90.9% presented with stage III disease. The median distance of tumour from the anal verge was 6.0 cm (range 1.0–11.0 cm).

### Preoperative treatment, surgery and postoperative chemotherapy

Cetuximab administration was discontinued after the first dose in four patients due to hypersensitivity reactions: these patients continued to receive capecitabine and radiotherapy. All 37 patients received the planned dose of radiation. Only two patients (5.4%) received less than 90% of the planned dose of capecitabine, one due to grade 3 hepatotoxicity and one due to grade 3 diarrhoea.

All 37 patients underwent surgery. Sphincter-sparing surgery was performed in 26 patients (70.3%) and abdominoperineal amputation in 11 patients (29.7%). One patient experienced back pain a few months after surgery and, on re-examination of the pretreatment MRI scan, was found to have had sacral bone metastasis prior to treatment. This patient was excluded from the survival and *KRAS* analyses. Tumour tissue from 32 patients was available for *KRAS* analysis and *KRAS* mutations were detected in the tumour tissue of 13 patients (40.6%).

Postoperative chemotherapy was administered to 34 patients. Thirty-two were able to receive all three planned cycles at the recommended dose: among these patients, one developed grade 3 leukopenia and one had grade 2 vomiting. The remaining two patients received two cycles of capecitabine due to the development of grade 2 diarrhoea.

### Treatment outcome

**FIGURE 1:**
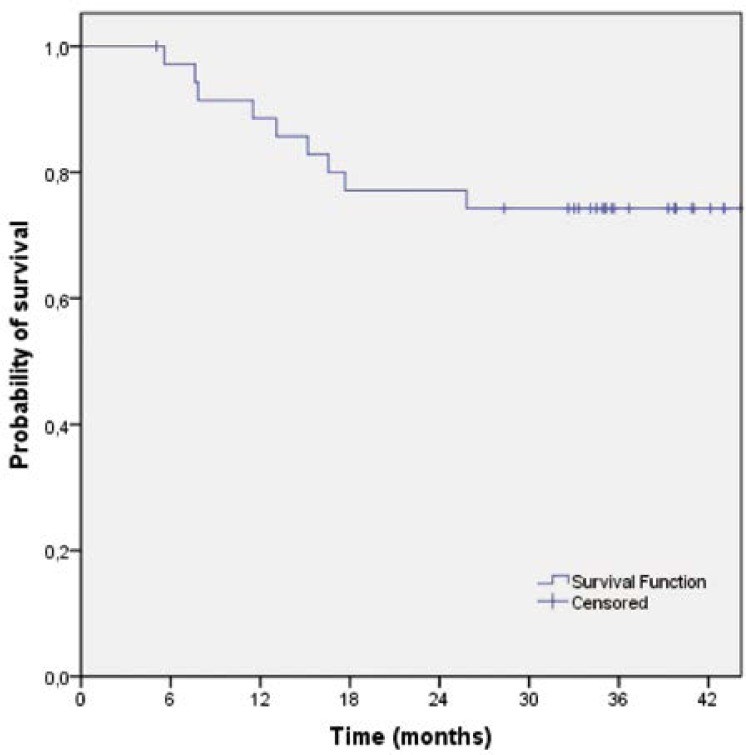
Relapse-free survival of patients treated with preoperative capecitabine-base chemotherapy and cetuximab (n=36).

**FIGURE 2:**
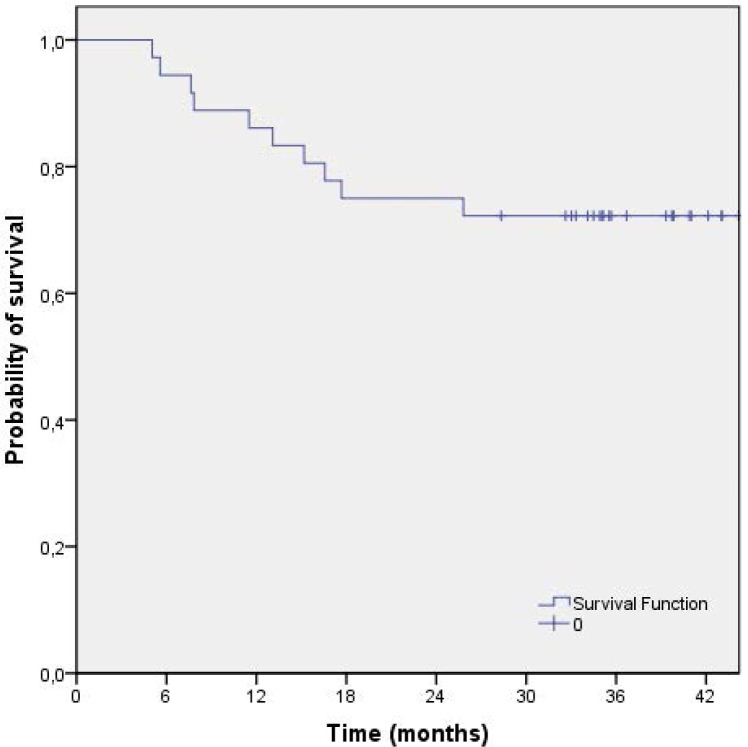
Disease-free survival of patients treated with preoperative capecitabine-base chemotherapy and cetuximab (n=36).

**FIGURE 3:**
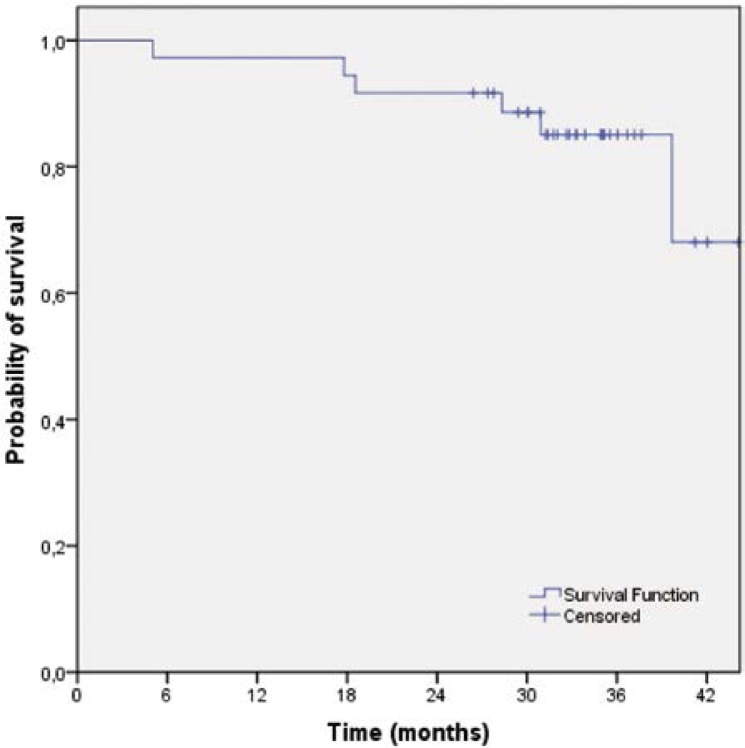
Overall survival of patients treated with preoperative capecitabine-base chemotherapy and cetuximab (n=36).

The median follow-up time for all 36 patients was 39.0 months (range 5.0–87.0 months). The median time to disease recurrence was 35.0 months (range 5.0–87.0 months). Local failure occurred in one patient (2.8%), 11.5 months after the end of treatment. The three-year local control rate was 96.9% (95% CI 90.9–100). Disease dissemination was observed in nine patients (25.0%) with the following pattern of distribution: liver (n=3; 8.3%), lung (n=3; 8.3%), suprarenal gland (n=1; 2.8%), bones (n=1; 2.8%) and synchronous lung plus retroperitoneum (n=1; 2.8%). The latest distant failure was observed 26 months after the end of treatment. No secondary malignancies were observed. The three-year relapse-free survival rate was 74.3% (95% CI 59.8–88.8) (Figure 1) and the three-year disease-free survival rate was 72.2% (95% CI 57.5–86.9) (Figure 2). The median survival has not yet been reached. The three-year survival rate was 68.1% (95% CI 36.7–99.4) (Figure 3).

There was no significant association between relapse- or disease-free survival and gender, age, tumour location in the rectum, type of surgery, pathological T or N status, TRG or tumour *KRAS* mutation status (Table 1).

### Deaths

As of September 2011, six of the 36 patients had died due to the following causes: rectal cancer (n=4), preoperative complications (n=1) and non-disease or treatment-related cause (accident at work) (n=1).

## Discussion

The results of this trial demonstrated that the addition of cetuximab to preoperative capecitabine-based CRT led to a pCR rate of 8%^8^ with long-term results showing a three-year local control rate of 96.9%, a three-year disease-free survival rate of 72.2% and a three-year overall survival rate of 68.1%. The treatment regimen was well tolerated. Since the results of the landmark randomized trial reported by the German Rectal Cancer Study Group in 2004, which confirmed the local control and reduced toxicity benefits of preoperative fluorouracil-based CRT over post-operative CRT for LARC^1^, ways to improve the efficacy of preoperative CRT regimens have been investigated. In the study conducted by the German Rectal Cancer Study Group, the preoperative treatment comprised 5 Gy (in fractions of 1.8 Gy per day, five days per week) and fluorouracil (1000 mg/m^2^/day 120-hour continuous infusion in the first and fifth weeks of radiotherapy). Four five-day cycles of fluorouracil (500 mg/m^2^) were administered one month after surgery. By five years, 6% of patients receiving preoperative therapy had relapsed.

In an earlier study from our group, we reported on the use of a capecitabine-based preoperative CRT regimen without cetuximab.^3^ The study used the same preoperative CRT regimen as that described in the present study but followed by four, rather than three, cycles of post-operative fluorouracil chemotherapy. Among 56 patients completing CRT and surgery, 55 had R0 resection. The pCR rate of 9.1% was similar to that reported in our current study with cetuximab and the five-year local control rate was 87.4% (95% CI 75.0–99.8%).

The original German Rectal Cancer Study Group trial reported a five-year overall survival rate of 76% for the use of preoperative CRT in LARC.^1^ In a recently reported randomized phase II study, the use of a dual chemotherapy combination of capecitabine and oxaliplatin in CRT followed by surgery and four cycles of post-operative capecitabine/oxaliplatin was reported to be associated with a three-year overall survival rate of 90%^23^, although the acute grade 3/4 toxicity associated with this regimen was fairly high: during CRT, 29% of patients experienced grade 3/4 toxicity, mainly diarrhea.^24^ The three-year overall survival results reported in our current study was 68.1%, which may in part reflect the single-agent composition of the chemotherapy component of the CRT regimen. In a phase II study in 40 patients, the addition of irinotecan to capecitabine and cetuximab in the preoperative CRT regimen was associated with a three-year overall survival rate of 94.7%.^17^

It is now well established that patients with mCRC who have tumour *KRAS* mutations are unlikely to benefit from the addition of cetuximab to standard chemotherapy.^25^,^26^ However, in rectal cancer, the influence of *KRAS* mutation status on tumour response in the presence of radiation is unclear. Data from a number of studies have suggested both no association^13^,^14^,^16^ and a negative association^27^ between the presence of tumour *KRAS* mutations and tumour regression and/or survival in patients with rectal cancer receiving preoperative CRT. In our study, no association between tumour *KRAS* mutation status and survival following preoperative cetuximab/capecitabine-based CRT could be found. Our findings support those from another analysis of data from 57 patients receiving CRT containing cetuximab plus irinotecan and capecitabine, in which tumour *KRAS* mutation status did not appear to influence tumour regression or disease-free survival.^15^ However, in a pooled analysis of data from 130 patients taking part in a number of phase II trials, including our own study, the presence of tumour *KRAS* mutations was significantly associated with pathologic nonresponse.^21^ It is possible, therefore, that tumour *KRAS* mutation status may be important in predicting outcome to cetuximab in this setting, but this would require larger scale analyses. Another study demonstrated that it was not *KRAS* mutation status that predicted achievement of a pCR following cetuximab-based neoadjuvant CRT, but rather the presence of *EGF* A+61G gene polymorphism, independent of *KRAS* mutation status.^22^ These data warrant additional investigation.

Our current study also reported relapse- and disease-free survival to be independent of gender, age, tumour location within the rectum, type of surgery, pathological T or N status and TRG, although, as for the *KRAS* analysis, the numbers of patients involved in the analysis are small. In a previous study, there was indication from a univariate analysis that post-operative parameters, including pathologic T or N status and TRG, were associated with disease-free survival.^28^ In multivariate analysis, however, only the association between pN and TRG and disease-free survival remained.

In conclusion, our study demonstrates that the addition of cetuximab to capecitabine-based preoperative CRT is feasible and is associated with an impressive three-year local control rate of 97%. The use of tumour *KRAS* mutation status as a biomarker to identify which patients are most likely to respond to cetuximab-based CRT requires further, larger scale investigation.

## Figures and Tables

**TABLE 1:** Univariate analysis of disease-free survival according to patient, disease and treatment characteristics

**Parameter**	**Chi-squared**	**Degrees of freedom**	p value*
**Gender**	0.010	1	0.919
**Age**	1.061	1	0.303
**Tumor location in the rectum** (lower, middle or upper third)	5.396	2	0.067
**Type of surgery** (abdominoperineal amputation or sphincter-sparing)	2.311	2	0.315
**Pathohistology**			
Node	0.275	2	0.872
Tumor	0.973	1	0.324
**Dworak regression grade**			
1–4	7.966	4	0.093
3–4 vs 0–2	0.254	1	0.614
**Tumour** KRAS **mutation status**	1.903	2	0.386

* Log-rank test. Number of patients = 36.
